# Three-dimensional dentoalveolar changes in open bite treatment in mixed dentition, spurs/posterior build-ups versus spurs alone: 1-year follow-up randomized clinical trial

**DOI:** 10.1038/s41598-022-15988-9

**Published:** 2022-07-20

**Authors:** Aron Aliaga-Del Castillo, Guilherme Janson, Lorena Vilanova, Lucia Cevidanes, Marilia Yatabe, Daniela Garib, Luis Ernesto Arriola-Guillén, Felicia Miranda, Camila Massaro, Silvio Augusto Bellini-Pereira, Antonio Carlos Ruellas

**Affiliations:** 1 Present Address: Department of Orthodontics and Pediatric Dentistry, School of Dentistry, University of Michigan, Ann Arbor, MI 48109 USA; 2 Department of Orthodontics, Bauru Dental School, University of São Paulo, Bauru, SP 17012901 Brazil; 3 Department of Orthodontics, Hospital for Rehabilitation of Craniofacial Anomalies, University of São Paulo, Bauru, SP 17012900 Brazil; 4 Division of Orthodontics and Division of Oral and Maxillofacial Radiology, School of Dentistry, Universidad Científica del Sur, 15067 Lima, Peru; 5 Department of Orthodontics, School of Dentistry, Federal University of Rio de Janeiro, Rio de Janeiro, 21941901 Brazil

**Keywords:** Health care, Medical research

## Abstract

This randomized clinical trial aimed to compare the three-dimensional dentoalveolar maxillary changes after anterior open bite treatment with bonded spurs and build-ups versus bonded spurs alone. Patients from 7 to 11 years of age with anterior open bite were randomly allocated into two groups. Bonded spurs and posterior build-ups were used in the experimental group and only bonded spurs were used in the comparison group. The randomization sequence was generated at www.randomization.com. Opaque, sealed and sequentially numbered envelopes were part of the allocation concealment. Digital dental models were acquired before (T1) and after 12 months of treatment (T2) and de-identified for analysis purposes. Three-dimensional changes of maxillary permanent incisors and first molars were evaluated by means of T1 and T2 dental model superimposition. Landmark-based registration on the posterior teeth and registration on the palate using regions of interest were performed. T or Mann–Whitney U tests were used for intergroup comparisons (*P* < 0.05). Mean difference (MD) and 95% confidence interval (CI) were calculated. Twenty-four children (17 girls and 7 boys) were included in the experimental group (mean age 8.22 ± 1.06 years) and 25 children (14 girls and 11 boys) were included in the comparison group (mean age 8.30 ± 0.99 years). After 12 months of treatment, inferior displacements of maxillary incisors were similar in the experimental (1.55–2.92 mm) and comparison (1.40–2.65 mm) groups. Inferior displacement of the maxillary molars was also similar in both groups (MD: − 0.13 mm; 95% CI − 0.38, 0.12). The experimental and comparison groups showed medial and lateral displacements of the permanent first molars, respectively (MD, − 0.31 mm; 95% CI − 0.51, − 0.11). Lingual inclination of the permanent first molars were observed in the experimental group and buccal inclination in the comparison group (MD, − 2.16°; 95% CI − 3.72, − 0.60). Similar three-dimensional displacements of maxillary central and lateral incisors, and inferior displacements of maxillary permanent first molars were observed in both groups. Bonded spurs associated with posterior build-ups demonstrated some medial displacement and lingual inclination of the maxillary permanent first molars while opposite changes were noticed in the comparison group.

Trial registration: Clinicaltrials.gov; NCT03702881, date of registration: October 11, 2018.

## Introduction

Anterior open bite is considered a challenging malocclusion to treat. It has a multifactorial etiology that includes genetic and environmental factors^1,2^. In children, anterior open bite is mostly caused by the presence of deleterious habits that break the equilibrium between anterior teeth and the muscles from the tongue and cheeks^2^. Some patients may have an associated vertical growth pattern that might influence the initial severity and treatment prognosis^2,3^.

Various treatment protocols have been reported to treat anterior open bite in the mixed dentition^4,5^. Removable plates with tongue cribs or fixed palatal cribs are the most common described to correct the habits^5–7^. The rapid molar intruder, posterior bite blocks and functional appliances as the open bite bionator and Frankel IV have been described for the control the vertical dimension^4,8–11^. Therapies associating interruption of the habits and control of the vertical growth have been also reported. Among these protocols, modified removable plate with tongue cribs and posterior bite block, fixed posterior bite blocks with tongue cribs; and palatal cribs, bonded spurs, or posterior bite blocks associated with vertical chincup or high-pull headgear have been studied^11–17^. In most of the cases, patient cooperation is imperative. Regarding bonded spurs, few randomized controlled trials (RCT) have been reported^7,18,19^ and only one included an associated therapy^19^.

Treatment changes in anterior open bite patients have been usually reported using lateral cephalograms^7,13–16,18,20,21^. Nevertheless, this type of evaluation has some inherent limitations due to the bi-dimensional (2D) assessment of three-dimensional (3D) structures^22^. No study has evaluated the dentoalveolar changes after anterior open bite treatment using 3D superimposition of digital dental models.

During anterior open bite treatment, significant vertical dentoalveolar changes in the anterior region are expected^6,7,12–16,18,20,21^. Consequently, structures of reference on the anterior region of the palate usually used to perform superimpositions are expected to show important vertical changes, as well^23,24^. Therefore, superimposition methods that do not consider structures in the most anterior region of the palate should be planned for treatment evaluation of anterior open bite^24^.

Some systematic reviews suggested the need of RCTs comparing different treatment modalities for anterior open bite malocclusion^4,5^. Recent RCTs have evaluated options associating removable and fixed posterior bite blocks with tongue cribs to correct the open bite by controlling the habits and the vertical development of posterior teeth^11,17^. Bonded spurs are a practical alternative to tongue cribs in children^7,18,21^, and posterior build-ups have been reported as effective in providing vertical control of posterior teeth by their bite block effect in adults^25^. Thus, the association of bonded spurs with posterior build-ups might be an alternative to be evaluated for open bite treatment in children. There is also need for further research on superimposition of digital dental models involving assessment of different procedures and treatment protocols^26^. This study aims to contribute in these issues, providing important information using 3D superimposition methods by comparing two protocols for anterior open bite treatment in the mixed dentition.

### Specific objectives or hypotheses

This study aimed to three-dimensionally compare the dentoalveolar maxillary changes after anterior open bite treatment with bonded spurs and build-ups versus bonded spurs alone by using 3D superimposition of digital dental models. The null hypothesis was that both protocols generate similar three-dimensional maxillary dentoalveolar effects.

## Methods

### Trial design and any changes after trial commencement

This study was planned as a secondary outcome analysis of a previous two-arm parallel single-center RCT with a 1:1 allocation ratio^19,27^. The Consolidated Standards of Reporting Trials (CONSORT) recommendations were followed^28^.

### Participants, eligibility criteria, and settings

Approval from the Ethics in Research Committee of Bauru Dental School, University of São Paulo, Bauru, Brazil (protocol: 68551617.8.0000.5417/2.112.035) was obtained. We confirm that all research was performed in accordance with relevant guidelines and regulations. Informed consent was obtained from all participants and their legal guardians. The research has been performed in accordance with the Declaration of Helsinki. The protocol of this study was registered at Clinicaltrials.gov (date of first registration: October 11, 2018, registration number: NCT03702881).

Recruitment of participants was performed from June 2017 to April 2018 at Bauru Dental School, University of São Paulo, Bauru, Brazil. Inclusion criteria consisted of children aged 7–11 years, anterior open bite greater than 1 mm (clinically evaluated as the vertical distance between the incisal edges of the maxillary and mandibular central incisors), fully erupted first permanent molars and central incisors, absence or mild anterior crowding and no need for maxillary expansion. For the younger participants, the vertical relationship between lateral and central incisors was considered to differentiate incomplete eruption from open bite. If the maxillary lateral incisors were closer to the occlusal plane compared to maxillary central incisors and the central incisors still showed a negative overbite (greater than 1 mm), the case was considered as open bite and the subject was eligible for treatment. This method was used based on previous reports^14,21,29^. If the maxillary lateral incisors are erupting, and the maxillary central incisors still have an open bite then it might indicate that an oral habit is impeding the eruption of the central incisors, since the maxillary lateral incisors usually erupt 1 year after the central incisors. Exclusion criteria comprised previous orthodontic treatment, syndromes or craniofacial anomalies, tooth agenesis and posterior crossbite.

All participants presented a history of at least one deleterious habit. Before recruitment, participants and their parents signed an informed consent.

### Interventions

**Figure 1:**
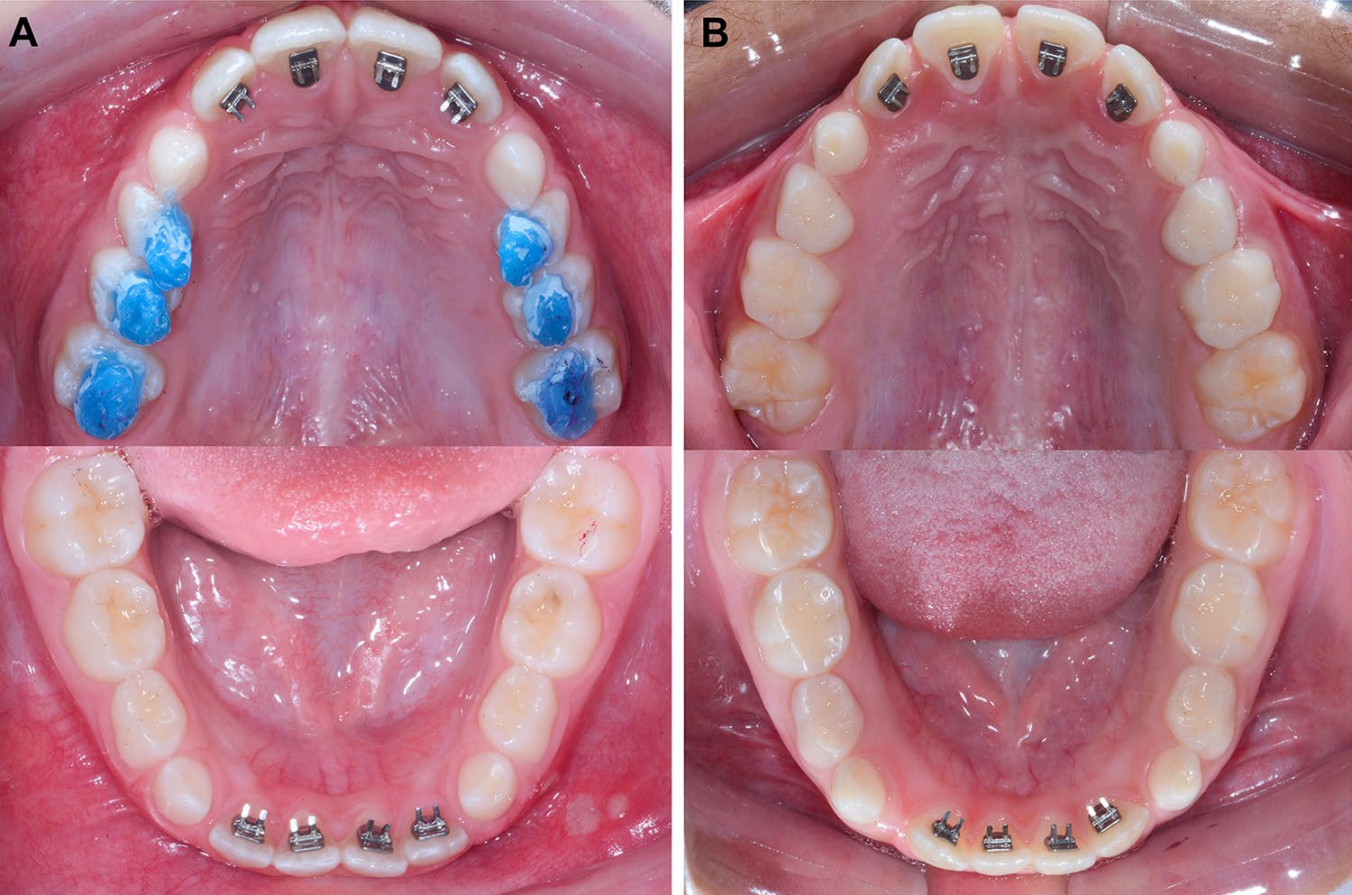
(**A**) Experimental group treated with bonded spurs and posterior build-ups. (**B**) Comparison group treated only with bonded spurs.

The experimental group was treated with spurs (Morelli Ortodontia, Sorocaba, São Paulo, Brazil) bonded at the cervical area of the palatal aspect of the maxillary incisors and at the incisal area of the lingual surface of the mandibular incisors^14,21^. Transbond XT adhesive was used (3M Unitek, Monrovia, CA, USA). All spurs were sharpened with a carborundum disk before bonding, as previously suggested^12^. In addition to the spurs, posterior build-ups of 2–3 mm resin blocks of light-cured orthodontic cement (Ortho Bite; FGM Dental Products, Joinville, Santa Catarina, Brazil) were bonded on the functional (palatal) cusps of the maxillary posterior teeth, as described in a previous study^25^ (Fig. 1A). The resin blocks were bonded first on the maxillary first permanent molars. Then, resin blocks were bonded on the other posterior teeth maintaining an occlusal forces balance^25^. The comparison group was treated only with spurs, bonded similarly as in the experimental group (Fig. 1B).

In cases of debonding or wearing, new spurs and build-ups were re-bonded, respectively, as promptly as possible. The evaluation follow-up was set at 12 months, based on previous reports^6,7,13–15,18,21^. After this period, the build-ups were removed in the experimental group and then, intraoral scanning was performed on the same day. The spurs were maintained in both groups as active retention for additional 12 months. Anterior open bite was considered corrected (closed) if the overbite was equal or greater than zero mm (zero: end-to-end vertical incisor relationship), as reported in previous studies in the mixed dentition^6,15^.

Digital dental models acquired by intraoral scanning (TRIOS3; 3Shape, Copenhagen, Denmark) were obtained before treatment (T1) and after the 12-month follow-up period (T2).

### Outcomes

The outcomes of the present study were the amount of three-dimensional dentoalveolar changes after superimposition (registration) of maxillary digital dental models. The primary outcome, overbite change, was previously evaluated^19,27^.

Previously reported registration methods were performed: landmark-based registration on the posterior teeth (TR) and registration on the palate using regions of interest (PR)^30^. Assessment of the medio-lateral, antero-posterior, superior-inferior, and complete 3D displacements were included in this trial. Additionally, the buccolingual inclinations and the mesiodistal angulations of maxillary incisors and permanent first molars were measured.

A three-dimensional analysis was performed with the 3D Slicer open-source software (Version 4.10.2; https://www.slicer.org). The following steps were performed.

#### Orientation

**Figure 2:**
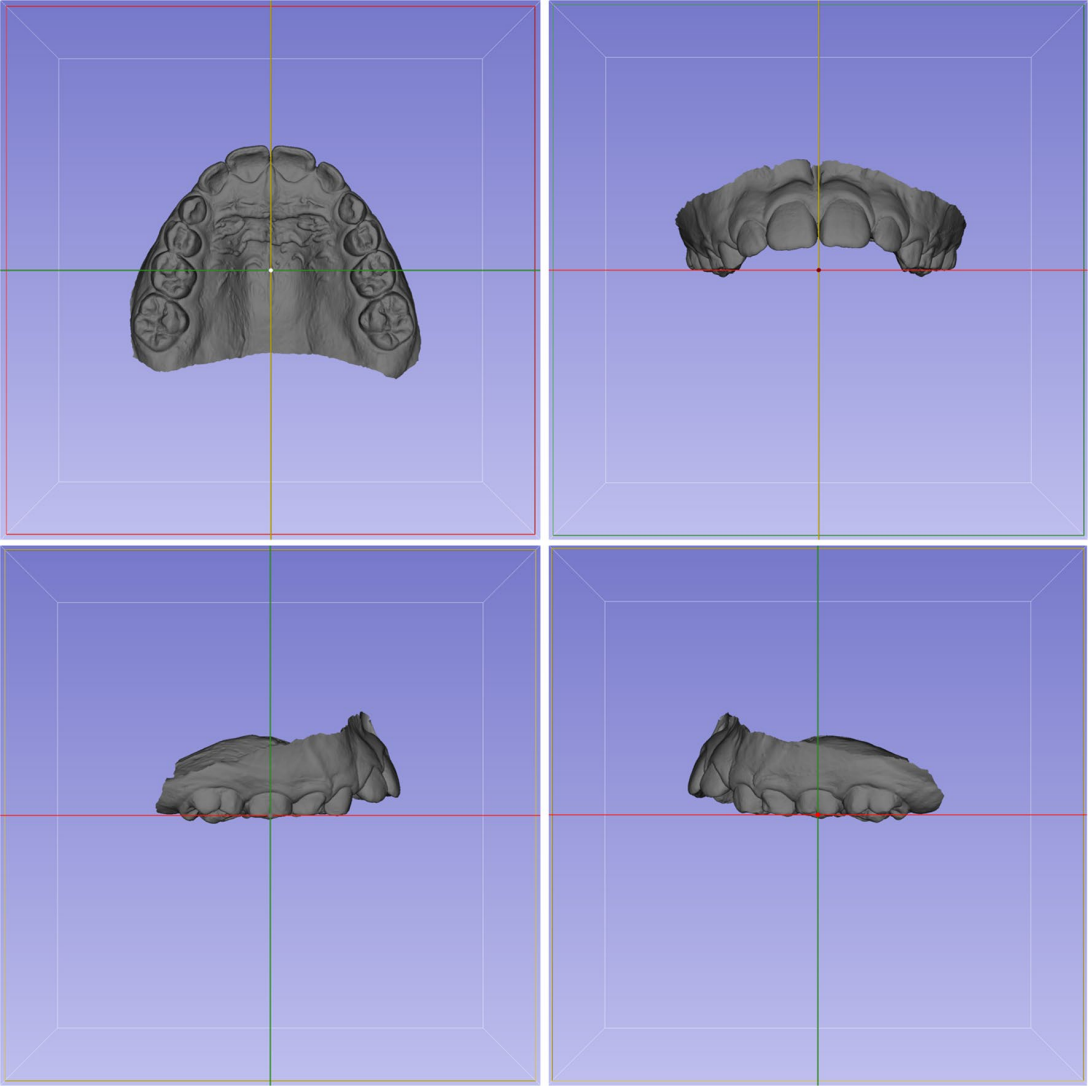
Study's methodology. 3D Orientation of the T1 digital dental model. Axial view (upper left): the midpalatal raphe was centered with the sagittal plane and the center of the buccal surface of the right deciduous second molar was positioned coincidently with the coronal plane. Sagittal view (lower right and left): the occlusal plane (passing through the mesiobuccal cusp tips of the permanent and deciduous first molars) was leveled with the axial plane on the right and left sides. Coronal view (upper right): the cusp tips of the right and left deciduous first molars were leveled with the axial plane.

A pre-established 3D coordinate system from 3D Slicer software was used to three-dimensionally orient all T1 maxillary dental models. The midpalatal raphe was centered with the sagittal plane and the center of the buccal surface of the right deciduous second molar was positioned coincidently with the coronal plane, in the occlusal view (Fig. 2). The occlusal plane (passing through the mesiobuccal cusp tips of the permanent and deciduous first molars) was leveled with the axial (horizontal) plane on the right and left sides, in the lateral view (Fig. 2). Finally, the cusp tips of the right and left deciduous first molars were leveled with the axial (horizontal) plane, in the frontal view (Fig. 2).

#### Approximation

**Figure 3:**
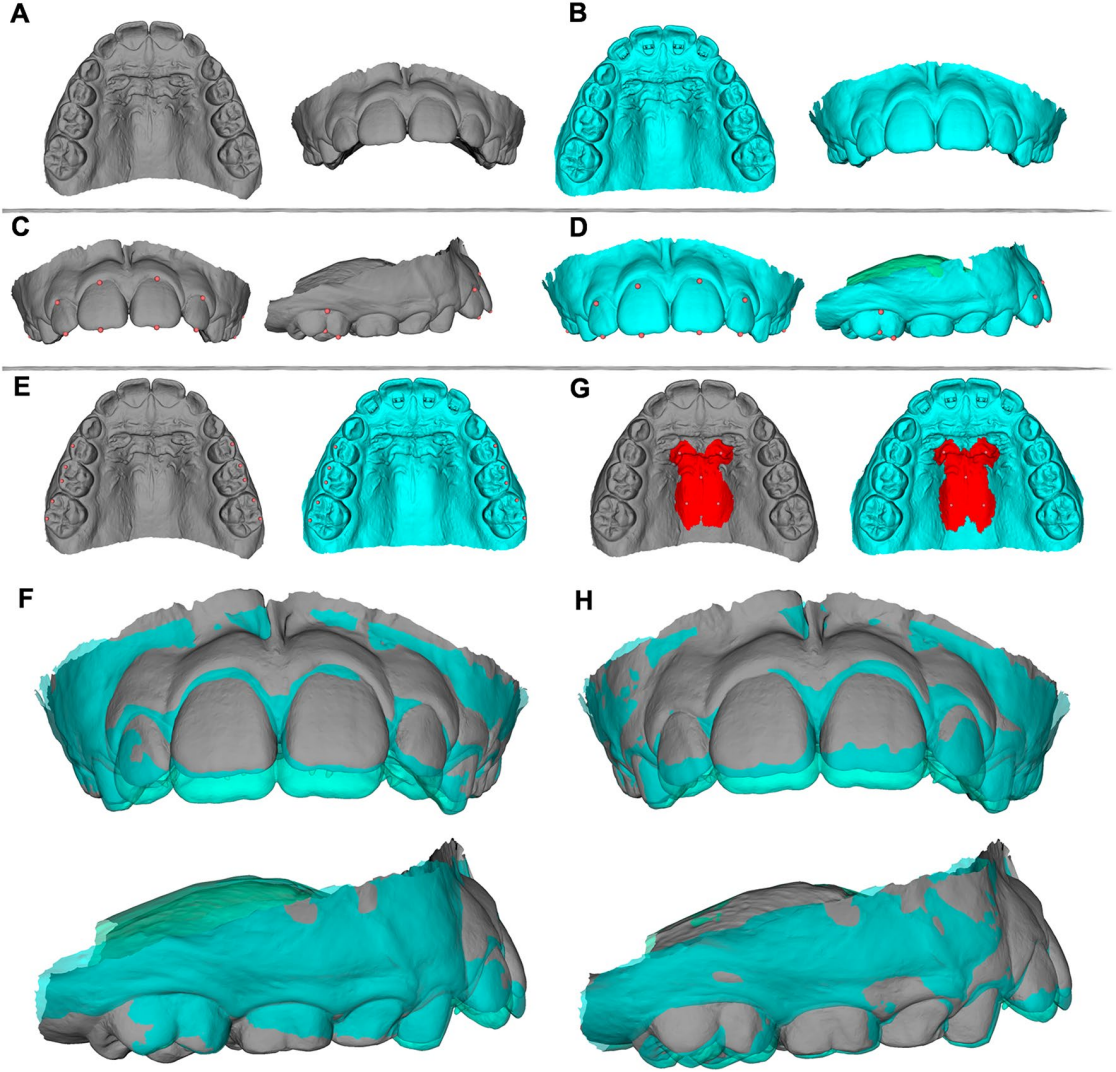
Study's methodology. (**A,B**) Pretreatment (T1, oriented) and posttreatment (T2 approximated to T1) digital dental models. (**C,D**) Landmarks placed on T1 (**C**) and T2 (**D**) models before registrations and that will be used for quantification of treatment changes. (**E**) Landmark-based registration on the posterior teeth. Landmarks were placed on T1 and T2 models only for registration. (**F**) Registered models after landmarks-based registration on the posterior teeth. (**G**) Registration on the palate using regions of interest. Regions of interest were created on T1 and T2 models for registration. (**H**) Registered models after registration on the palate using regions of interest.

Landmarks placed on the mesiobuccal cusp tips of the permanent first molars and deciduous second molars on the right and left sides were used to approximate the T2 dental models to the oriented T1 models (Fig. 3A). These landmarks were not used to obtain measurements. Then, the “approximatedT2” dental model was obtained (Fig. 3B).

#### Landmarks placement (pre-labeling)

Landmarks were placed on the mesiobuccal cusp tip and on the occlusal and cervical limits of the buccal groove of the permanent first molars; on the middle of the incisal edge and at the most cervical limit of the buccal surface of the lateral and central incisors, bilaterally on the “oriented T1” (Fig. 3C) and on the “approximated T2” dental models (Fig. 3D). Files containing the landmarks for each model were created for each patient. Since these landmarks were used for measurements, this step was performed previously to the registration steps to use the same landmarks placed on the “approximated T2” dental model for the two methods of registration, preventing any landmark identification error^31^.

#### Registrations

##### Landmark-based registration on the posterior teeth (TR)

New specific landmarks used for registration were placed on the distal and mesiobuccal cusp tips of the permanent first molars and deciduous second molars, and on the mesiobuccal cusp tip of the deciduous first molar on the right and left sides, on the “oriented T1” and “approximatedT2” dental models (Fig. 3E). The “TR” T2 registered model (Fig. 3F) and the corresponding registration matrix were created after models´ registration using the fiducial registration tool.

##### Registration on the palate using regions of interest (PR)

One central landmark was placed on the midpalatal raphe and was positioned coincident with the coronal plane. Two anterior and two posterior lateral landmarks were placed on the angle between the most superior and lateral surfaces of the palatal concavity on the right and left sides. The anterior and posterior limits were defined by two horizontal lines perpendicular to the midpalatal raphe, one passing through the middle of the occlusal surface of the deciduous first molar and the other passing between the permanent first and deciduous second molars, respectively. Regions of interest of 10 and 15 mm were created around the lateral and central landmarks, respectively (Fig. 3G) on the “oriented T1” and on the “approximated T2” model. The “PR” T2 registered model (Fig. 3H) and the corresponding registration matrix were created after models´ registration using the ROI registration tool.

#### Application of the registration matrix to the pre-labeled landmarks

The matrix created from the TR and PR registrations were applied to the landmark (pre-labeled landmarks) files that were previously created for the “approximatedT2” model (step # 3). Each T2 registered model had its corresponding landmarks file. This procedure aimed to use the same landmarks on the two registered models, preventing any landmark identification error^31^.

#### Measurements

The landmark (pre-labeled landmarks) files of the oriented T1 model (step # 3) and TR and PR T2 registered models (step # 5) were used to obtain the measurements. Medio-lateral (x coordinate), antero-posterior (y coordinate), superior-inferior (z coordinate) and 3D displacements, buccolingual inclination and mesiodistal angulation changes between oriented T1 and T2 registered models were obtained using the Q3DC tool. Positive values denoted lateral/anterior/inferior displacements, buccal inclination and mesial angulation. Negative values denoted medial/posterior/superior displacements, lingual inclination and distal angulation. The means between the right and left sides were considered for the analyses.

### Sample size calculation

Sample size was calculated considering a significance level of 5%, a test power of 80%, and a difference to be detected between groups of 1.5 mm in the overbite change (primary outcome) with a standard deviation of 1.69 mm^14^. The minimal sample size required was 21 participants per group.

### Interim analyses and stopping guidelines

Not applicable.

### Randomization (random number generation, allocation concealment, implementation)

The random number generation was performed at the Randomization.com website (http://www.randomization.com) using random block sizes guaranteeing an equal distribution in the groups^32^. Allocation concealment included sequentially numbered, opaque and sealed envelopes. The envelopes contained the cards with the assigned treatment and were inserted into foil to increase opacity. All the envelopes were prepared before trial commencement. The name and initial characteristics of the patients were written on the external surface of the envelope. The envelopes were torn open instead of being unsealed. Different persons independently performed the random number generation, allocation concealment and implementation^33^.

### Blinding

Since the operator and patients were aware about the appliances that were installed, double blinding was not plausible. Nevertheless, all digital dental models were de-identified before analysis and the three-dimensional evaluation was blinded^34^.

### Statistical analyses

The same examiner performed the three-dimensional analysis of 16 patients (30% of the sample) twice with a one-month interval between assessments. The intraexaminer reliability was evaluated with the intraclass correlation coefficient.

The SPSS software (Version 25; IBM, Armonk, NY, USA) was used to perform the statistical analyses. Normal distribution of the data was evaluated with the Shapiro–Wilk test. Intergroup comparisons were performed using t or Mann–Whitney U tests. Statistical significance was set at *P* < 0.05.

## Results

### Participant flow

**Figure 4:**
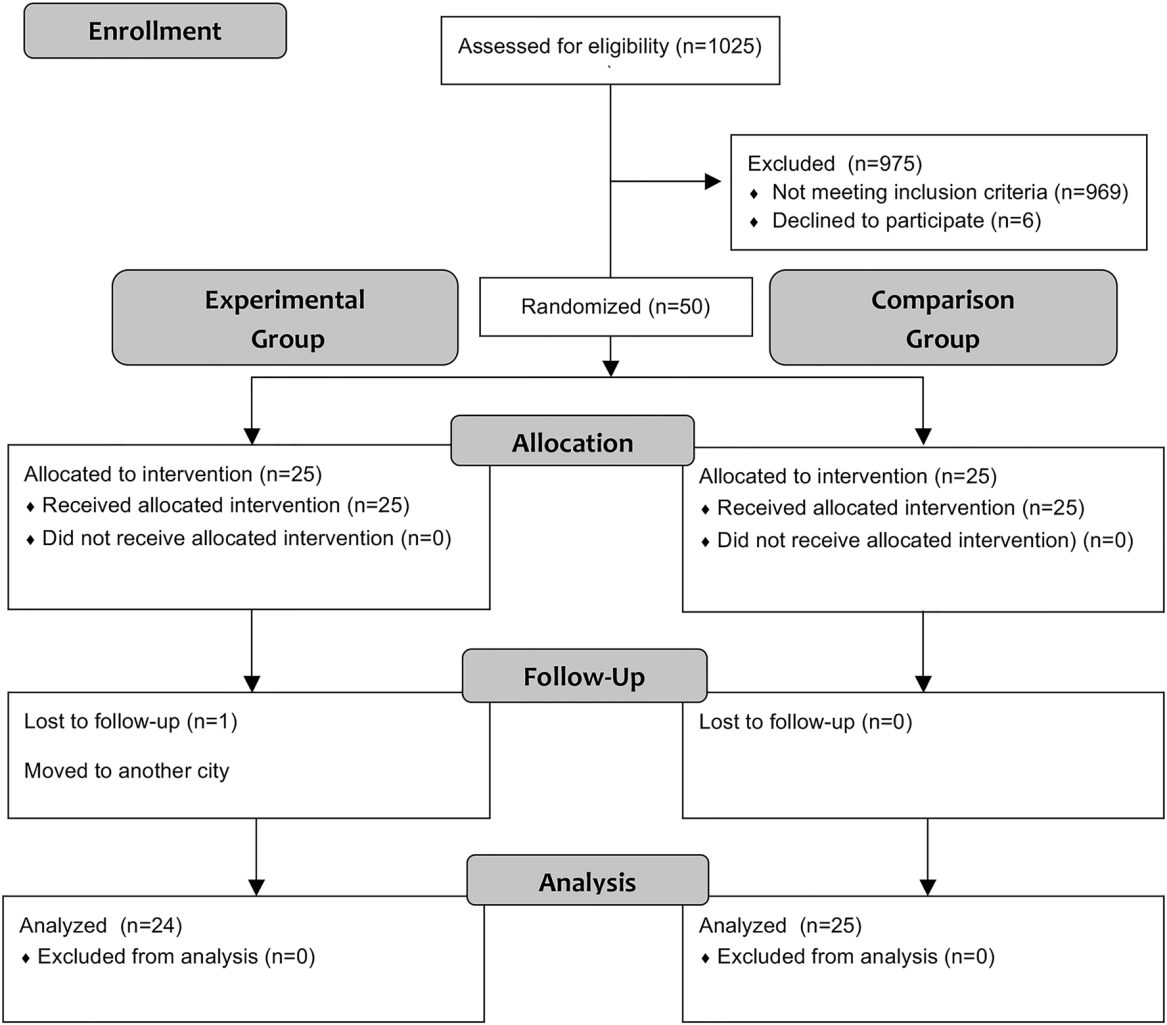
Consolidated standards of reporting trials flow diagram^19^.

One thousand and twenty-five children were assessed for eligibility during recruitment (June 2017 – April 2018); 969 did not meet the inclusion criteria and were excluded and 6 declined to participate. Fifty patients were randomized in a 1:1 ratio (Fig. 4).

### Baseline data

The initial age and sex distribution were similar between groups (Table 1). In addition, they showed a similar initial amount of anterior open bite and similar mandibular plane angle and lower anterior face height. They showed similar skeletal vertical and sagittal growth pattern (Table 1).

**Table 1:** Initial age, sex distribution, and T1 and T2 data.

	Experimental group (n = 24)		Comparison group (n = 25)		Mean difference	95% CI (mean difference)		*P* value
	Mean	SD	Mean	SD				
Age (years)	8.22	1.06	8.30	0.99	− 0.08	− 0.67	0.51	0.787^‡^
**Sex**								
Female	n = 17	70.8%	n = 14	56.0%	–	–	–	
Male	n = 7	29.2%	n = 11	44.0%	–	–	–	0.377*
**Cephalometric variables**								
Overbite T1(mm)	− 4.45	1.49	− 4.36	1.65	− 0.09	− 1.00	0.82	0.843^‡^
Overbite T2 (mm)	0.39	1.42	0.48	1.57	− 0.09	− 0.95	0.77	0.830^‡^
Overjet T1 (mm)	3.70	1.90	3.81	1.94	− 0.11	− 1.22	0.99	0.839^‡^
Overjet T2 (mm)	3.87	1.78	3.76	1.47	0.11	− 0.82	1.05	0.806^‡^
SN.GoGn T1 (°)	34.47	5.29	34.42	4.34	0.05	− 2.73	2.82	0.865^†^
SN.GoGn T2 (°)	34.30	4.91	34.10	5.02	0.19	− 2.66	3.05	0.936^†^
LAFH T1 (mm)	56.11	3.42	56.92	4.33	− 0.81	− 3.06	1.44	0.472^‡^
LAFH T2 (mm)	56.66	3.59	57.66	4.52	− 1.00	− 3.35	1.35	0.398^‡^
SNA T1 (°)	83.19	2.67	83.65	3.89	− 0.46	− 2.38	1.47	0.636^‡^
SNA T2 (°)	82.55	2.60	83.30	4.14	− 0.75	− 2.74	1.23	0.447^‡^
SNB T1 (°)	79.00	3.40	79.50	3.06	− 0.50	− 2.35	1.36	0.594^‡^
SNB T2 (°)	78.84	3.11	79.82	3.50	− 0.98	− 2.89	0.93	0.305^‡^
ANB T1 (°)	4.20	1.79	4.14	1.81	0.06	− 0.98	1.09	0.914^‡^
ANB T2 (°)	3.70	1.59	3.46	1.90	0.24	− 0.77	1.25	0.629^‡^

^‡^t test; *Fisher exact test; ^†^Mann Whitney U test; SD, Standard deviation; SN.GoGn, mandibular plane angle; LAFH, lower anterior face height; SNA, sella-nasion- Apoint angle; SNB, sella-nasion-Bpoint angle; ANB, Apoint-Nasion-Bpoint angle.

### Number analyzed for each outcome, estimation, and precision

One patient from the experimental group moved to another city and was lost during the follow-up. A per-protocol basis was considered for the analysis and included 24 patients in the experimental and 25 patients in the comparison group, respecting their original group assignment.

The intraclass correlation coefficient values (Supplementary Table S1) demonstrated very good to excellent intraexaminer reliability and ranged from 0.941 (95% CI 0.840–0.979) to 1.000 (95% CI 1.000–1.000)^35^.

Although improvements were observed after 12-month follow-up for all patients, anterior open bite was corrected in 16 patients (66.7%) from the experimental group and in 18 patients (72%) from the comparison group^19^. Anterior open bite was considered corrected (closed) if the overbite was equal or greater than zero mm^6,15^. After 12 months, the spurs were maintained in place for all patients. They acted as active retention in patients that showed a corrected anterior open bite, and continued as active treatment in patients that improved but remained with some anterior open bite.

Significant intergroup differences for the angulations of maxillary lateral incisors and linear displacements and inclination of maxillary molars were observed (Tables 2 and 3). The experimental group showed greater distal angulation of the maxillary lateral incisor than the comparison group (Tables 2 and 3). The maxillary molar showed medial displacement in the experimental group (− 0.18 mm) and lateral displacement in the comparison group (0.13 mm) (MD, − 0.31 mm; 95% CI − 0.51, − 0.11; *P* value, 0.003) (Table 3). Significantly smaller anterior and 3D displacements of maxillary molars were observed in the experimental group (0.62 mm and 1.04 mm, respectively) compared to the comparison group (0.94 mm and 1.36 mm, respectively) (Anterior displacement MD, − 0.32 mm; 95% CI − 0.60, − 0.03; *P* value, 0.029. 3D displacement MD, − 0.32 mm; 95% CI − 0.56, − 0.08; *P* value, 0.005) (Table 3). The experimental group showed lingual inclination of the maxillary molar (− 1.33°), while buccal inclination was observed in the comparison group (0.83°) (MD, − 2.16°; 95% CI − 3.72, − 0.60; *P* value, 0.018) (Table 3). Similar inferior displacements of maxillary molars were observed in the experimental (0.43 mm) and comparison group (0.56 mm) (MD, − 0.13 mm; 95% CI − 0.38, 0.12; *P* value, 0.305) (Table 3).

**Table 2:** 3D Changes using the landmark-based registration on the posterior teeth (TR).

	Experimental group (n = 24)		Comparison group (n = 25)		Mean difference	95% CI (mean difference)		*P* value
	Mean	SD	Mean	SD				
**Medio-lateral displacement**								
Mx.1	− 0.25	0.40	− 0.26	0.38	0.01	− 0.22	0.23	0.660^†^
Mx.2	0.07	0.44	− 0.09	0.52	0.16	− 0.15	0.47	0.318^†^
**Antero-posterior displacement**								
Mx.1	− 0.02	0.99	− 0.25	0.82	0.24	− 0.28	0.76	0.365^‡^
Mx.2	0.29	0.69	0.04	0.65	0.25	− 0.17	0.67	0.241^‡^
**Supero-inferior displacement**								
Mx.1	2.92	0.99	2.65	1.11	0.27	− 0.33	0.88	0.366^‡^
Mx.2	2.40	0.97	2.00	0.87	0.41	− 0.18	0.99	0.169^‡^
**3D displacement**								
Mx.1	3.17	0.99	2.88	1.08	0.29	− 0.30	0.89	0.329^‡^
Mx.2	2.63	0.90	2.23	0.79	0.40	− 0.13	0.94	0.134^‡^
**Buccolingual inclination**								
Mx.1	− 6.31	4.26	− 6.15	3.54	− 0.16	− 2.41	2.09	0.887^‡^
MX.2	− 6.07	4.97	− 3.90	4.15	− 2.17	− 5.05	0.71	0.318^†^
**Mesiodistal angulation**								
Mx.1	0.55	3.25	− 0.03	2.36	0.58	− 1.04	2.21	0.474^‡^
Mx.2	− 3.16	3.08	− 0.35	3.71	− 2.81	− 5.01	− 0.61	0.014^‡^*

^†^Mann Whitney U test; ^‡^t test; SD, Standard deviation; Mx.1, maxillary central incisor; Mx.2, maxillary lateral incisor; (−) values denote medial/posterior/superior displacements, lingual inclination and distal angulations; (+) values denote lateral/anterior/inferior displacements, buccal inclination and mesial angulation.

*Statistically significant at *P* < 0.05.

**Table 3:** 3D changes using the registration on the palate through regions of interest (PR).

	Experimental group (n = 24)		Comparison group (n = 25)		Mean difference	95%CI (mean difference)		*P* value
	Mean	SD	Mean	SD				
**Medio-lateral displacement**								
Mx.1	− 0.26	0.40	− 0.26	0.37	0.01	− 0.22	0.23	0.653^†^
Mx.2	0.05	0.44	− 0.05	0.60	0.09	− 0.25	0.43	0.579^‡^
Mx.6	− 0.18	0.41	0.13	0.29	− 0.31	− 0.51	− 0.11	0.003^‡^*
**Antero-posterior displacement**								
Mx.1	0.42	0.80	0.46	0.59	− 0.04	− 0.44	0.37	0.509^†^
Mx.2	0.82	0.55	0.77	0.51	0.05	− 0.29	0.38	0.775^‡^
Mx.6	0.62	0.41	0.94	0.57	− 0.32	− 0.60	− 0.03	0.029^‡^*
**Supero-inferior displacement**								
Mx.1	1.94	0.78	1.88	1.10	0.06	− 0.49	0.61	0.836^‡^
Mx.2	1.55	0.80	1.40	0.79	0.15	− 0.36	0.66	0.553^‡^
Mx.6	0.43	0.38	0.56	0.47	− 0.13	− 0.38	0.12	0.305^‡^
**3D displacement**								
Mx.1	2.29	0.81	2.26	0.93	0.03	− 0.47	0.53	0.900^‡^
Mx.2	2.05	0.69	1.94	0.62	0.10	− 0.31	0.52	0.615^‡^
Mx.6	1.04	0.37	1.36	0.46	− 0.32	− 0.56	− 0.08	0.005^†^*
**Buccolingual inclination**								
Mx.1	− 3.57	3.48	− 3.68	3.56	0.11	− 1.92	2.13	0.914^‡^
Mx.2	− 3.23	4.51	− 1.56	4.05	− 1.67	− 4.38	1.04	0.293^†^
Mx.6	− 1.33	2.92	0.83	2.50	− 2.16	− 3.72	− 0.60	0.008^‡^*
**Mesiodistal angulation**								
Mx.1	0.55	3.23	− 0.02	2.36	0.57	− 1.05	2.20	0.479^‡^
Mx.2	− 3.02	2.95	− 0.42	3.64	− 2.60	− 4.74	− 0.46	0.018^‡^*
Mx.6	3.07	2.91	2.93	3.57	0.13	− 1.75	2.01	0.888^‡^

^†^Mann Whitney U test; ^‡^t test; SD, Standard deviation; Mx.1, maxillary central incisor; Mx.2 maxillary lateral incisor; Mx.6, maxillary first molar; (−) values denote medial/posterior/superior displacements, lingual inclination and distal angulation; (+) values denote lateral/anterior/inferior displacements, buccal inclination and mesial angulation.

*Statistically significant at *P* < 0.05.

### Harms

All participants and their parents were oriented to spit out the spurs and/or build-ups in case of debonding. They were aware that, eventually, debonded spurs could be swallowed or aspirated into the lungs. No aspiration nor swallow episode was reported for any participant. Debonding rates were 2.5% for spurs and 5.3% for build-ups. New spurs and build-ups were bonded again, promptly. Although some discomfort was reported during the beginning of the treatment, patients showed good adaptability overall.

Maintenance of adequate oral hygiene was instructed for all participants. Nevertheless, some participants showed plaque accumulation around the spurs during the follow-up period.

## Discussion

### Main findings in the context of the existing evidence and interpretation

The two treatment protocols evaluated in this study similarly improved the anterior open bite initial condition after 12 months of treatment. The amount of overbite correction was previously reported to be greater than 4 mm in both groups^19^. Success rates of 66.7% and 72% were observed for the experimental and comparison groups, respectively^19^. Three-dimensional superimposition of maxillary dental models in the present study provided important and objective information about individual tooth movement, using previously validated methods^36^. Maxillary dental model registration is usually performed using the palatal rugae and surrounding structures as references^26,36–39^. This investigation focused on three-dimensional outcomes comparing anterior open bite early treatment protocols and using superimposition of maxillary digital dental models.

Treatment effects of anterior open bite malocclusion in the mixed dentition show important dentoalveolar vertical development of the anterior region^6,11,17^. This is caused as a consequence of the elimination of interferences between the maxillary and mandibular incisors mostly caused by habits^6,7,11,13–15,17,18,20,21^. Similar inferior displacements and lingual inclinations were observed for the maxillary incisors. These changes played an important role in anterior open bite correction, as previously reported in other recent studies^11,17^. Vertical changes could be expected at the first rugae and incisive papilla in growing patients and these changes were related with positional changes of the maxillary incisors^23,24^. Therefore, superimposition methods excluding the most anterior region of the palate should be used, even more in open bite patients because the vertical positional changes in this area, with treatment, could affect the superimposition results^23,24,30^.

In this study, the most anterior region of the palate was excluded. Landmark-based registration on the posterior teeth (TR) and registration on the palate using regions of interest (PR) were used (Fig. 3E,G). These methods have been previously assessed and showed adequate reproducibility^30^. TR superimposition showed the relative changes of the anterior teeth in relation to the posterior teeth and PR superimposition showed the relative changes of the anterior and posterior teeth in relation to the palate. Both registration methods were performed to show the amount of changes on the incisors and on the maxillary permanent first molars that could be expected, using different references for superimposition. TR superimposition should be indicated when no significant changes are expected on the molars, for interim/short-term assessments or when the palate region cannot be used for superimposition because of the presence of fixed appliances or when the structures are not adequately scanned or reproduced. PR superimposition could be used for longer follow-ups assessments and needs adequate reproduction of the palate.

The PR and TR superimpositions showed slight differences for the antero-posterior displacement of the maxillary central incisors. The PR showed smaller values for inferior displacement, 3D displacement and lingual inclination of the incisors (Tables 2 and 3, Fig. 3F,H). The differences in the amounts of displacements and inclinations of the incisors between the TR and PR methods could be expected because the methods used different structures for superimposition^30^.

Inferior displacements of the maxillary incisors were similar in both groups and ranged from 1.55 mm (PR) to 2.92 mm (TR) in the experimental group and from 1.40 mm (PR) to 2.65 mm (TR) in the comparison group (Tables 2 and 3). These effects were expected as a response of spurs effects, as previously reported in cephalometric studies^7,14,15,18,19,21^. Inferior displacement was greater in the central than in the lateral incisors. These findings were expected because the central incisors are the most affected in anterior open bite malocclusion.

A greater distal angulation of the maxillary lateral incisors was observed for the experimental group (Tables 2 and 3). The canine eruption path and process and the muscular balance might have influenced this result. However, they were not evaluated in this study. This difference was smaller than 2.82°, it does not have a significant impact on anterior open bite correction or extrusion of anterior teeth, and may not have clinical significance.

The different medio-lateral displacement of the maxillary permanent first molars observed in the groups could be explained because of the presence of build-ups in the experimental group (Table 3). Because the build-ups were bonded on the palatal functional cusps of the maxillary permanent first molars, the occlusal forces could lead to a relative medial displacement and lingual inclination of these teeth. This has been previously speculated when build-ups are used for open bite correction in adults and should be considered during treatment planning^25^. Conversely, the maxillary permanent first molars in the comparison group were free and showed lateral displacement (Table 3).

The significantly smaller anterior or mesial displacement of the maxillary permanent first molar observed in the experimental group could also be attributed to the presence of build-ups (Table 3). Posterior build-ups demonstrated an effect in producing some anterior control of molars. Although these variables showed significant differences, the mean intergroup differences were smaller than 0.33 mm. Thus, no clinically significant implication could be expected. Additionally, leeway space availability during the late mixed dentition also might have interfered with mesial movement of maxillary molars in some patients.

Inferior displacements of the permanent first molars ranged from 0.43 to 0.56 mm for the experimental and comparison groups, respectively. Due to the presence of posterior build-ups in the experimental group, control of inferior displacement (extrusion) of the maxillary permanent first molars was expected, in this group. However, minimal and nonsignificant differences between groups were observed (Table 3). This study findings indicate that posterior build-ups may have some control on lateral displacement of the maxillary permanent first molars but no control on inferior displacement of these teeth. A previous cephalometric study focused on the dentoskeletal changes of this sample found smaller extrusion of maxillary molars in the group treated with bonded spurs and build-ups compared to spurs alone. However, that finding was not enough to produce an important counter-clockwise rotation of the mandible^19^. This could be related to the normal growth potential that children at this age range present^13–16^. No significant control of inferior displacement of the maxillary molars were also reported in some studies on other associated therapies aimed to control the vertical dimension in children^13–16^. Recent RCTs have been reported involving posterior bite blocks and cribs in patients with similar ages^11,17^. One study^11^ compared the removable posterior bite blocks associated with cribs versus the anterior open bite bionator. Both treatment approaches were aimed to produce vertical control of posterior teeth; however, extrusion of maxillary molars was observed with the two therapies as well in our study. The authors^11^ mentioned that the expected effect of a more limited extrusion of posterior teeth should be compared with an untreated control group. On the other hand, another RCT evaluated fixed posterior bite blocks associated with cribs and included and untreated control group in their comparisons. They showed that the proposed associated therapy led to an intrusion of the maxillary molars while an extrusion was observed in the untreated controls^17^. These results might be related to the appliance´s design. Further comparisons involving different treatment approaches, untreated controls and using 3D superimposition of maxillary digital dental models are needed to compared our results with.

The experimental group showed significantly smaller 3D displacement of the maxillary permanent first molar than the comparison group (Table 3). This was expected because 3D displacement considers the medio-lateral, anterior–posterior, and superior–inferior displacements as a combination. So, the experimental group had smaller 3D displacement because of the smaller medial and anterior displacements compared to the comparison group. However, the differences between groups was smaller than 0.33 mm and might not have a clinically significant impact.

The maxillary first molars showed lingual inclination in the experimental group and buccal inclination in the comparison group (Table 3). This explains the medial and lateral displacements observed for the experimental and comparison groups, respectively. As mentioned before, these differences could be expected due the presence of build-ups on the molars of the experimental group^25^. A previous study in this sample reported a slight decrease of the intermolar distance in the group treated with bonded spurs and builds-ups^27^. Associating the results of this study, it could be suggested that posterior build-ups produces lingual inclination of the maxillary molars that reflects on a slight decrease of the maxillary intermolar distance.

The present study showed similar displacements of the maxillary central and lateral incisors in both groups. Opposite medio-lateral displacement and buccolingual inclination of the maxillary permanent first molars were observed between groups. In the experimental group, the first molars showed lingual movement (medial displacement and lingual inclination) while buccal movement (lateral displacement and buccal inclination) was noted in the comparison group. Future evaluations of 3D dentoalveolar changes should be performed in treated, untreated and normal occlusion children. This will help to understand what 3D changes should be attributed to treatment or growth.

### Limitations

The absence of an untreated control group was the main limitation of this study. This would allow understanding of the three-dimensional dentoalveolar maxillary changes in untreated anterior open bite patients due to facial growth. However, this group was not possible due to ethical reasons^7^.

In this specific study, an end-to-end vertical incisor relationship (zero mm) or a positive overbite was established as a parameter to consider correction. This was previously reported in similar studies in the mixed dentition^6,15^. This means that the anterior open bite was closed but does not mean that an ideal overbite has been achieved. After 12 months, spurs were maintained in patients with closed open bites as active retention and in some patients, that remained with an anterior open bite to continue correction. Vertical skeletal involvement at pretreatment, short-term follow-up and oral habits persistence have been reported as related factors to this condition^7,14,21^. Future studies with longer follow-up periods and assessment of the long-term stability of the outcomes should be performed. In addition, patient´s perception during treatment should be evaluated.

### Generalizability

Our results should be only generalized to patients with similar age range and initial anterior open bite characteristics contemplated in this study.

## Conclusions

- Bonded spurs associated with posterior build-ups and bonded spurs alone showed similar three-dimensional displacements of the maxillary central and lateral incisors, after 12-month treatment;
- The maxillary permanent first molars showed medial displacement and lingual inclination with bonded spurs associated with posterior build-ups. Conversely, lateral displacement and buccal inclination of the maxillary permanent first molars was observed using only bonded spurs. Vertical displacements of maxillary permanent first molars were not significantly different between the two groups.
- Both treatment protocols were similarly effective for anterior open bite correction (66.7% and 72% success rates after 12-month treatment, for bonded spurs associated with posterior build-ups and bonded spurs alone, respectively).
- Bonded spurs associated with posterior build-ups did not produce greater vertical control than bonded spurs alone after 12-month treatment.

## Supplementary Information

Supplementary Table S1.

## Data Availability

The data analyzed during the current study are available from the corresponding author on a reasonable request.
