# Supplementary material for: Three-dimensional dentoalveolar changes in open bite treatment in mixed dentition, spurs/posterior build-ups versus spurs alone: 1-year follow-up randomized clinical trial
Source: Sci Rep. 2022 Jul 20;12:12378. doi: 10.1038/s41598-022-15988-9 (PMC9300740; doi:10.1038/s41598-022-15988-9)
Supplement: Supplementary file 1 — Supplementary Table S1. [file 41598_2022_15988_MOESM1_ESM.pdf]

Three-dimensional dentoalveolar changes in open bite treatment in mixed dentition, spurs/posterior build-ups versus spurs alone: one-year follow-up randomized clinical trial

Aron Aliaga-Del Castillo, Guilherme Janson, Lorena Vilanova, Lucia Cevidanes, Marilia Yatabe, Daniela Garib, Luis Ernesto Arriola-Guillén, Felicia Miranda, Camila Massaro, Silvio Augusto Bellini-Pereira, Antonio Carlos Ruellas.

Supplementary Table S1. Intraexaminer reliability results.

	Intraclass Correlation Coefficient	95% Confidence Interval	
		Lower Bound	Upper Bound
**Landmark-based registration on the posterior teeth (TR)**			
**Medio-Lateral Displacement**			
**Mx.1**	1.000	1.000	1.000
**Mx.2**	0.996	0.987	0.999
**Antero-Posterior Displacement**			
**Mx.1**	0.997	0.993	0.999
**Mx.2**	0.994	0.983	0.998
**Supero-Inferior Displacement**			
**Mx.1**	0.999	0.996	0.999
**Mx.2**	0.999	0.996	1.000
**3D Displacement**			
**Mx.1**	0.999	0.996	0.999
**Mx.2**	0.999	0.996	1.000
**Buccolingual Inclination**			
**Mx.1**	0.999	0.998	1.000
**Mx.2**	1.000	0.999	1.000
**Mesiodistal Angulation**			
**Mx.1**	1.000	1.000	1.000
**Mx.2**	1.000	1.000	1.000
**Registration on the palate using regions of interest (PR)**			
**Medio-Lateral Displacement**			
**Mx.1**	1.000	1.000	1.000
**Mx.2**	0.999	0.996	1.000
**Mx.6**	1.000	1.000	1.000
**Antero-Posterior Displacement**			
**Mx.1**	0.993	0.981	0.998
**Mx.2**	0.989	0.967	0.997
**Mx.6**	0.979	0.942	0.993
**Supero-Inferior Displacement**			
**Mx.1**	0.985	0.958	0.995
**Mx.2**	0.985	0.953	0.995
**Mx.6**	0.999	0.998	1.000
**3D Displacement**			
**Mx.1**	0.985	0.959	0.995
**Mx.2**	0.979	0.936	0.993
**Mx.6**	0.941	0.840	0.979
**Buccolingual Inclination**			
**Mx.1**	0.996	0.987	0.998
**Mx.2**	0.997	0.992	0.999
**Mx.6**	1.000	1.000	1.000
**Mesiodistal Angulation**			
**Mx.1**	1.000	1.000	1.000
**Mx.2**	0.999	0.997	1.000
**Mx.6**	0.996	0.987	0.998

Mx.1, maxillary central incisor; Mx.2 maxillary lateral incisor; Mx.6, maxillary first molar
